# Love in the Shadow of Oblivion: The Meaning Given to Love in Couples Living With Alzheimer’s Disease, From a Family View

**DOI:** 10.1093/geroni/igaa057.1482

**Published:** 2020-12-16

**Authors:** Orit Shavit, Aaron Ben-Ze’ev, Israel (Issi) Doron

**Affiliations:** 1 Haifa University, zichron Yaacov, Hefa, Israel; 2 University of Haifa, Haifa, Israel

## Abstract

Aims and objectives: To deeply understand the significance of love between spouses who live with Alzheimer’s Disease (AD) in a familial aspect. Background: While there is extensive empirical knowledge on the subject of AD, as well as about love, little is known about love in old age, and even less is known about love between spouses who live with AD. This study is a pioneering study that describes love and relationship with AD. Design: A phenomenological qualitative research, which enables a close examination of the experience that accompanies the couple and their adult children from a family perspective that has not yet been examined. It belongs to the stream of Social Constructivism whereby the purpose of interviewing more than one family member was used to capture the process by which family members construct their identity as individuals and as a family unit. Methods: Forty-five in-depth interviews were conducted with n = 15 triads including the couple and their adult child, based on Interpretative Phenomenological Analysis (IPA). Results: Three central themes emerged: (1) The meaning of AD, (2) Continuity and discontinuity of love prior and with AD, (3) The meaning of love in coupled living with AD. Conclusions: Commitment with AD is a moral-ethical obligation, and not necessarily because of love, due to the increasing price of separation. Future research is discussed. Key words: Alzheimer’s disease, Love, Relationship, Meaning, couples

